# Hepatic Macrophages in Liver Injury

**DOI:** 10.3389/fimmu.2020.00322

**Published:** 2020-04-17

**Authors:** Zhao Shan, Cynthia Ju

**Affiliations:** ^1^Center for Life Sciences, School of Life Sciences, Yunnan University, Kunming, China; ^2^Department of Anesthesiology, McGovern Medical School, University of Texas Health Science Center at Houston, Houston, TX, United States

**Keywords:** Kupffer cells, monocyte-derived macrophages, liver injury, microenvironmental cues, cellular crosstalk

## Abstract

Ample evidence suggests that hepatic macrophages play key roles in the injury and repair mechanisms during liver disease progression. There are two major populations of hepatic macrophages: the liver resident Kupffer cells and the monocyte-derived macrophages, which rapidly infiltrate the liver during injury. Under different disease conditions, the tissue microenvironmental cues of the liver critically influence the phenotypes and functions of hepatic macrophages. Furthermore, hepatic macrophages interact with multiple cells types in the liver, such as hepatocytes, neutrophils, endothelial cells, and platelets. These crosstalk interactions are of paramount importance in regulating the extents of liver injury, repair, and ultimately liver disease progression. In this review, we summarize the novel findings highlighting the impact of injury-induced microenvironmental signals that determine the phenotype and function of hepatic macrophages. Moreover, we discuss the role of hepatic macrophages in homeostasis and pathological conditions through crosstalk interactions with other cells of the liver.

## Introduction

Hepatic macrophages, consisting of liver resident Kupffer cells (KCs) and monocyte-derived macrophages (MoMϕs), play a central role in maintaining homeostasis of the liver as well as contributing to the progression of acute or chronic liver injury (1). Kupffer cells are the most abundant tissue macrophages in mammalian bodies, accounting for 80–90% of total tissue macrophages (2). In the liver, KCs are distributed along the hepatic sinusoids, preferentially near the periportal areas (3). In the healthy liver, a small number of MoMϕs are located mainly in the portal triad areas (4, 5). In mouse, KCs and MoMϕs can be distinguished by their differential expression of certain cell surface markers. Kupffer cells are defined as CD11b^low^, F4/80^hi^, C-type lectin domain family 4 member F, CD68^+^, and CX3C chemokine receptor 1 (CX_3_CR1)^−^ (6, 7). MoMϕs are CD11b^+^, F4/80^+^, Ly6C^+^, macrophage colony-stimulating factor 1 receptor (CSF1R)^+^. Human KCs are not as well-characterized and normally identified as CD68^+^ cells (8). Human MoMϕs are normally identified as CD14^+^, CC-chemokine receptor 2 (CCR2)^+^ cells (Table 1) (8, 9).

**Table 1:** Difference between KCs and MoMϕs.

	**KCs**	**MoMϕs**
Origins	Fetal liver-derived erythromyeloid progenitors	Circulatory monocytes from bone marrow-derived haematopoietic stem cells
Location	Along the hepatic sinusoids, preferentially near the periportal area	Mainly in the portal triad of the healthy liver
Cell surface markers in mice	CD11b^low^, F4/80^hi^, Clec4F^+^, CD68^+^, CX3CR1^−^	CD11b^+^, F4/80^+^, Ly6C^+^, CSF1R^+^
Cell surface markers in humans	CD68^+^	CD14^+^, CCR2^+^
Functional differences	Induce immune tolerance to innocuous antigens; defend against pathogens; sense alterations in tissue integrity and maintain tissue homeostasis; orchestrate tissue repair after injury; facilitate leukocyte recruitment	Contribute to anti-bacterial responses; replenish KCs after injury; co-existence of subsets with opposing functions of pro-inflammatory and anti-inflammatory; pro-fibrogenic and anti-fibrogenetic

*KCs, Kuppfer cells; MoMϕs, Monocytes-derived macrophages; CD11b, cluster of differentiation molecule 11B; Clec4f, C-type lectin domain family 4 member f; CX3CR1, CX3C chemokine receptor 1; CSF1R, Colony-stimulating factor 1 receptor; CCR2, Chemokine (C-C motif) receptor 2*.

A salient function of macrophages is to remove pathogens and dead cells (3). In a healthy liver, KCs constantly phagocytose aged erythrocytes, neutrophils, and effector CD8^+^ T cells, thereby maintaining homeostasis within the liver (10–12). Monocyte-derived macrophages are also able to phagocytose dead endothelial cells and maintain integrity of vasculature under normal condition (13). The phagocytosis function of macrophages and its contribution to liver homeostasis and disease have been recently highlighted in an excellent review (14). Aside from phagocytosis, macrophages act as antigen-presenting cells and regulate adaptive immune responses. It is known that KCs play a central role in inducing immune tolerance to innocuous antigens that reached the liver from the intestine (15). In contrast, MoMϕs that are recruited into the liver lose the tolerogenic phenotype and instead contribute to antigen-specific proinflammatory immune responses (Table 1) (16). During liver injury, a large number of circulating monocytes infiltrate into the liver and further differentiate into MoMϕs (17). Injury-induced modulations of the liver tissue microenvironment, such as soluble mediators released by activated/stress cells and accumulation of dead cells, influence the phenotypes of both KCs and MoMϕs and determine their involvement in aggravation of liver injury or restoration of liver functions (6, 16, 17). Under healthy and disease conditions, we call any signals received from local microenvironment and prompted functional differentiation of hepatic macrophages as microenvironmental signals. Understanding how microenvironmental signals affect the heterogeneity of hepatic macrophages and how hepatic macrophages interact with other cells during liver injury is instrumental not only in gaining basic knowledge but also in designing therapeutic strategies to treat liver diseases. In this review, we summarize the latest findings about specific environmental signals that impact the phenotype and function of hepatic macrophages and discuss the functions of hepatic macrophages in healthy and diseased liver through interacting with other cells.

## Origin of Hepatic Macrophages

The recent development and improvement of research tools have allowed us to gain novel insights into the origin of hepatic macrophages. Contrary to the notion that KCs are originated solely from bone marrow–derived circulating monocytes (18), recent evidence suggests that KCs are mainly derived from yolk sac erythromyeloid progenitors (EMPs). Erythromyeloid progenitors colonize the fetal liver at E8.5 and give rise to macrophage precursors (pMacs) at E9.5 in a CX3CR1-dependent manner. A distinct transcriptional programming involving the upregulation of DNA binding 3 (Id3) is important in the further differentiation of pMacs into KCs (19). With regard to the maintenance of tissue-resident macrophages including KCs, an interesting “niche competition” model has been put forth. This model proposes that bone marrow–derived circulating monocytes and EMPs have an almost identical potential to develop into KCs and that they compete for a restricted number of niches. However, during liver development, the majority of liver niches are taken by EMPs and few by monocytes. At steady state, the niches in liver are self-maintained and tightly controlled by specific Maf transcription repressors and enhancers (20). However, when a significant number of KCs die or depleted experimentally, circulating monocytes have been shown to contribute to the KCs pool, suggesting that circulating monocytes have the potential as their embryonic counterparts to develop into KCs (21–23).

Monocyte-derived macrophages are derived from circulatory monocytes, which are developed from bone marrow–resident hematopoietic stem cells (23). Hematopoietic stem cells first differentiate into common lymphoid progenitors and then into granulocyte–monocyte progenitors (24, 25). Granulocyte–monocyte progenitors further give rise to common monocyte progenitors (cMoPs) under the regulation of macrophage CSF-1 (26). Eventually, cMoPs differentiate into circulatory monocytes. These distinct steps of hepatic stellate cell (HSC) differentiation into monocytes are tightly regulated by a number of transcription factors and epigenetic DNA methylation mechanisms (23). In mice, there are two subsets of monocytes in the liver: Ly6C^hi^ and Ly6C^low^ monocytes. The Ly6C^hi^ monocytes can differentiate into Ly6C^low^ monocytes, which can further differentiate into MoMϕs (27, 28). In humans, the CD14^hi^CD16^−^ and CD14^−^CD16^hi^ monocytes correlate with Ly6C^hi^ and Ly6C^low^ monocytes in mice, respectively (29).

## Impact of Tissue Microenvironmental Factors on Hepatic Macrophages

Irrespective of the cellular origins of hepatic macrophages, they differentiate into different phenotypes depending on the stimulatory signals in the tissue environment (30). In disease conditions, there are two major types of stimulatory signals (Table 2), including danger-associated molecular patterns (DAMPs) and pathogen-associated molecular patterns (PAMPs).

**Table 2:** Microenvironment in the liver.

	**Examples**	**Reported in liver diseases**	**References**
DAMPs	HMGB-1	AILI, I/R injury, NASH	(31–36)
	HSP-70	AILI	(37, 38)
	Bile acids	Cholestatic liver disease	(39–43)
	Histones	I/R injury	(44)
	FFAs	NASH	(45)
	mtDNA	NASH	(45)
PAMPs	Bacteria-derived products, e.g. LPS	ALD, NASH	(46–49)
	Hepatitis virus, e.g. HBV, HCV, HEV	Hepatitis virus infection	(50–52)
Phagocytosis of cellular debris	Apoptotic cells	LPS/D-gal-induced hepatitis, CCL4-induced liver fibrosis, ALD	(53–55)

*DAMPs, danger-associated molecular patterns; PAMPs, pathogen-associated molecular patterns; HMGB-1, high mobility group box 1; HSP-70, heat shock protein 70; FFAs, free fatty acids; mtDNA, mitochondrial DNA; LPS, Lipopolysaccharides; HBV, hepatitis B virus; HCV, hepatitis C virus; HEV, hepatitis E virus; AILI, Acetaminophen-induced liver injury; I/R, Ischemia/Reperfusion; NASH, Non-alcoholic steatohepatitis; ALD, Alcoholic liver disease*.

Danger-associated molecular patterns are a diverse group of molecules including nucleic acid in various conformations (e.g., single-/double-stranded RNA or DNA), nuclear proteins [e.g., high mobility group box 1 (HMGB1)], cytosolic proteins [e.g., heat shock protein 70 (HSP-70)], purine nucleotides [e.g., adenosine triphosphate (ATP)], or mitochondrial compounds (e.g., mtDNA, N-formyl peptides). Danger-associated molecular patterns are recognized by pathogen recognition receptors (PRRs), which are expressed on hepatic macrophages and can activate the cells (56, 57). These PRR receptors include Toll-like receptors (TLRs), nucleotide-binding oligomerization domain–like receptors (NLRs), and retinoic acid-inducible gene 1–like receptors (56). Danger-associated molecular patterns play a critical role in activating macrophages during sterile liver injury, which can be caused by a variety of stimuli, such as overdose of acetaminophen (APAP), cholestatic obstruction, excess accumulation of free fatty acids (FFAs), or hepatic ischemia, and reperfusion (I/R) (31, 58). The release of DAMPs is common in these situations, and it may be an active process from viable or dying cells, or a passive process leaking out from necrotic cells (56).

In APAP-induced liver injury (AILI), hepatocyte damage leads to the release of HMGB1, which activates KCs to produce proinflammatory cytokines and chemokines (31). High mobility group box 1 is a nuclear-binding protein that can function as a DNA chaperone in nucleus, sustaining autophagy in cytosol and DAMP molecule outside the cell (32). High mobility group box 1–deficient mice show less inflammation and neutrophil recruitment post-APAP treatment when compared to their wild-type counterparts (31, 33). Mice lacking the receptor for advanced glycation end products, a receptor for HMGB1, on bone marrow–derived cells display a similar phenotype as the HMGB1-deficient mice (31, 33). Together, these data indicate that HMGB1 activates KCs and contributes to AILI (31, 33). Aside from HMGB1, HSP-70 has also been reported to be released by necrotic hepatocytes during AILI (37). Heat shock protein 70 is an important component of cellular machinery, facilitating protein folding, but also an inducible stress-response protein that can undergo translocation to the cell surface or is released into the extracellular milieu during cellular stress or necrotic death (38). Heat shock protein 70 can stimulate macrophages to promote immune response and inflammation (37).

Similar to the necroinflammatory injury pattern in AILI, cholestatic liver disease is also characterized by hepatocytes necrosis (59). Lesions in bile duct canaliculi cause an increase of bile acids, which result in liver injury, but appear to drive KCs into anti-inflammatory state (39, 59). Bile acids can bind to G-protein–coupled bile acid receptor (TGR5), which is expressed on hepatic macrophages (40). Activation of TGR5 in macrophages reduces proinflammatory cytokines while maintaining anti-inflammatory cytokines (41). The anti-inflammatory effect of TGR5 on macrophages is mediated by inhibiting nuclear factor κB and JNK signaling pathways and NLRP3 inflammasome (41, 43).

Hepatic I/R injury is another example of sterile tissue injury in which DAMPs are released. It is reported that after I/R injury hepatocytes actively secrete HMGB1, which binds to TLR4 and activates KCs (34). In addition to HMGB1, other DAMPs, such as histones, DNA fragments, ATP, and mitochondrial reactive oxygen species (ROS), also activate KCs via different PRRs, such as TLR9 (recognizing histones) (44), TLR3 (recognizing RNA) (60, 61), TLR4 (recognizing HSP-70) (62), and nucleotide-binding domain leucine-rich repeat containing family pyrin domain containing 3 (NLRP3) (recognizing ATP) (63, 64). As a result, KCs secrete ample proinflammatory cytokines to exacerbate I/R injury (65).

Non-alcoholic steatohepatitis (NASH) is characterized by steatosis with tissue injury and inflammation (35). Increased accumulation of fat in hepatocytes leads to lipotoxicity, which induces apoptotic and necrotic death of hepatocytes with concomitant release of DAMPs, such as HMGB1 and FFAs (35). Released HMGB1 activates KCs via TLR4 as in other liver injury cases (35, 36). Free fatty acids decrease the mitochondrial membrane potential and subsequently induce mtDNA release from the mitochondria to the cytoplasm (45). Released mtDNA causes NLRP3 inflammasome activation in KCs, which further promotes the production of proinflammatory interleukin 1β (IL-1β) (45).

Pathogen-associated molecular patterns include pathogen-specific polysaccharides, lipoproteins, and nucleic acids. They bind to PPRs expressed on hepatic macrophages and trigger inflammatory activation of these cells (66). In alcoholic liver disease (ALD), excess chronic ingestion of alcohol increases gut permeability and causes translocation of bacteria-derived products, such as lipopolysaccharides (LPSs), from the gut to the liver (67). Lipopolysaccharide binds to CD14 in association with TLR4 on KCs to initiate proinflammatory signaling (46). Numerous studies have demonstrated that KCs play a central role in alcohol-induced inflammation and injury of the liver (27, 28). Kupffer cells produce cytokines such as tumor necrosis factor α (TNF-α) and free radicals to promote ALD (68, 69). In addition to KCs, MoMϕs can be differentiated from the circulating monocytes that are recruited into the liver as a result of the inflammatory response during liver injury (28). Two types of MoMϕs (Ly6C^low^ MoMϕs and Ly6C^hi^ MoMϕs) have been reported to coexist in the liver of mice after chronic alcohol feeding. The Ly6C^low^ MoMϕs exhibit restorative phenotype, whereas Ly6C^hi^ MoMϕs exhibit proinflammatory phenotype. A large dose of alcohol binge after chronic alcohol feeding enhances liver inflammation and injury with an increased ratio of Ly6C^hi^/Ly6C^low^ MoMϕ. Moreover, Ly6C^hi^ MoMϕ switches to Ly6C^low^ MoMϕ upon phagocytosis of apoptotic hepatocytes. Taken together, KCs and MoMϕ are involved in the pathogenesis of ALD (27, 28, 70).

Whereas, translocation of LPS from the gut lumen to portal circulation in ALD may result from direct alcohol toxicity to intestinal epithelium (67), increased exposure to intestinal LPS has also been considered in the pathogenesis of NASH (71). Increased levels of LPS in the portal circulation have been observed during NASH development (72). The role of LPS-TLR4 signaling in non-alcoholic fatty liver disease (NAFLD) has been demonstrated in a number of studies (47). Toll-like receptor 4 expression on KCs is elevated both in patients with NASH and mice fed with methionine–choline–deficient diet (47, 48). Depletion of KCs by clodronate liposomes significantly reduced TLR4 expression and steatohepatitis (47). Together, endotoxin appears to play an important role in NAFLD through TLRs on KCs, particularly TLR4. However, the function of MoMϕ in response to endotoxin during NAFLD remains unclear.

Hepatitis virus infection is a leading cause of chronic liver diseases (49). Hepatitis B virus (HBV) modulates liver macrophage functions to favor viral replication and survival. These modulations include reduced production of antiviral cytokine IL-1β and enhanced levels of immunosuppressive IL-10 (73). Hepatitis C virus (HCV) can enter KCs through phagocytic uptake. Subsequently, viral RNA triggers myeloid differentiation primary response gene 88 (MyD88)–mediated TLR7 signaling to induce IL-1β expression. Hepatitis C virus uptake concomitantly induces a potassium efflux that activates the NLRP3 inflammasome and promotes the secretion of activated IL-1β. Interleukin 1β produced by KCs contributes to HCV disease severity (74). It has also been reported that the function of monocytes/macrophages is impaired in pregnant patients with hepatitis E virus (HEV)–induced acute hepatitis (50). Toll-like receptor 3 and TLR7 expressions are reduced, and MyD88 downstream molecules, IRF3 and IRF7, are decreased in macrophages from these patients (50). The macrophage phagocytic activity and *Escherichia coli*–induced ROS production are significantly impaired in macrophages from HEV patients as well (50). These data suggest that the impact of viruses on hepatic macrophages could result in immune suppression and/or inflammation and tissue damage.

### Phagocytosis of Dead Cells

A salient function of macrophages is to remove dead cells and cellular debris, which is a key initial step in resolving tissue inflammation and promoting tissue repair from injury. It has been widely reported that phagocytosis of dead cells triggers transcriptional reprogramming of macrophages to switch from a proinflammatory to an anti-inflammatory and tissue restorative phenotype. For example, it is shown that the membrane-bound transforming growth factor β (TGF-β) on apoptotic cells can trigger KCs to produce IL-10, which suppresses the proinflammatory immune response in a mouse model of LPS/d-galactosamine–induced hepatitis (51, 52). In a mouse model of ALD, it is reported that phagocytosis of cellular debris triggers hepatic macrophages to switch from proinflammatory to an anti-inflammatory phenotype (28). Similarly, in a mouse model of carbon tetrachloride–induced liver fibrosis, phagocytosis of cellular debris induces Ly6C^hi^ proinflammatory, profibrotic MoMϕs to differentiate into antifibrotic macrophages (52). The restorative macrophages are the most abundant subset in the liver during maximal fibrosis resolution and represent the predominant source of matrix metalloproteinases. Moreover, it has been demonstrated that phagocytosis of necrotic hepatocytes by macrophages activates Wnt ligand secretion, thereby promoting liver regeneration (53).

In summary, the particular function of KCs and MoMϕ is tightly regulated by microenvironmental signals, such as DAMPs and PAMPs. The same signals may play a similar role in different liver diseases; for example, HMGB1 released by hepatocytes triggers KC activation in AILI, I/R, and NASH, indicating similar functions of macrophages in various liver diseases (32–34, 37, 54, 56, 58, 75, 76). In certain liver diseases, such as NAFLD and ALD, DAMPs and PAMPs coexist to regulate macrophage functions (35, 56, 77, 78) However, more studies are warranted to better understand the differential functions of KCs and MoMϕs in a given disease situation.

## Communication Between Hepatic Macrophages and Other Cells in the Liver

There are five major cell types in the liver including hepatocytes, biliary epithelial cells (cholangiocytes), liver sinusoidal endothelial cells (LSECs), KCs, and HSCs (66, 79). Hepatocytes account for roughly 80% of the liver's mass (79), and they perform a number of vital functions, including protein synthesis, detoxification, and metabolism of lipids and carbohydrates (79). Cholangiocytes are another type of epithelial cells in the liver, lining the lumen of the bile ducts (79). Liver sinusoidal endothelial cells form the lining of the smallest blood vessels in the liver, part of the reticuloendothelial system (79). Liver sinusoidal endothelial cells regulate the passage of molecules from the blood vessels into the liver (79). Hepatic stellate cells are found in the space of Disse, between hepatocytes and LSECs. Hepatic stellate cells can transdifferentiate from a quiescent state to an activated, highly proliferative, and wound-healing myofibroblast (80). In addition, there are a variety of other immune cells such as monocytes, neutrophils, and platelets that contribute to liver homeostasis as well as disease progression (66). In different types and stages of liver diseases, hepatic macrophages are critically involved in the progression and regression via close communication with other cells in the liver (Table 3) (108).

**Table 3:** The crosstalk of hepatic macrophages with other cells in the liver.

	**Interacted cell types**	**Roles of hepatic macrophages in interaction**	**Reported in liver diseases**	**References**
KCs	Hepatocytes	KCs activate Notch signaling in hepatocytes	ICC	(81)
	Hepatocytes	KCs are essential for proliferation of liver progenitor cells	Choline-deficient, ethionine-supplemented diet-mediated liver injury	(82)
	Hepatocytes	KCs trigger hepatocytes senescence	ALD	(83)
	Hepatocytes	KC are activated by hepatocytes-derived extracellular vesicles	ALD	(84, 85)
	HSCs	KCs activate HSCs and modulate fibrogenic responses in HSCs	Liver fibrosis	(86, 87)
	HSCs	KCs secrete IFN-γ to induce apoptosis of HSCs	Liver fibrosis	(88)
	HSCs	HSCs induce KCs differentiation and decrease its cytokine secretion	Liver fibrosis	(89)
	LSECs	KCs are essential for LSECs' uptake of hyaluronic acid	*In vitro* culture	(90)
	MoMϕs	KCs produce CCL2 to recruit MoMϕs	Amodiaquine-induced liver injury	(91)
	Neutrophils	The production of TNF-α and TGF-β by KCs is promoted by neutrophil-secreted IL-17; Express adhesion molecules to recruit neutrophils	Cholestatic liver injury, LPS-induced liver injury	(92, 93)
	NKT cells	KCs produce IL-1β to recruit and activate NKT cells	Alcoholic steatosis	(94)
	CD4^+^ T cells	KCs produce ROS, IL-6 and TNF-α to recruit CD4^+^ T cells.	Hepatic I/R injury	(95)
	T cells	KCs produce IL-10, TGF-β, ROS, IDO, PGE2/J2 to induce and maintain T cell tolerance or apoptosis	Liver transplantation, HBV infection, *in vitro* culture	(96–101)
	CD8^+^ T cells	KCs prime CD8^+^ T cells to differentiate into effector cells to kill viruses	HBV infection	
	Platelets	KCs promote adhesion of platelets on the KCs to encase the bacteria and facilitate anti-bacterial responses	Bacteria infection in the liver	
MoMϕs	cholangiocytes	MoMϕs release IL-6 to promote the proliferation of cholangiocytes	Cholestatic liver disease	(102, 103)
	cholangiocytes	MoMϕs are recruited by cholangiocytes-derived osteopontin and MCP-1	Partial Hepatectomy	(104)
	LSECs	LSECs are activated by MoMϕs	Partial Hepatectomy	(105)
	NKT cells	MoMϕs promote NKT cells over-activation and cell death	NAFLD	(106)
	NKT cells	MoMϕs produce IL-12 to activate NKT cells, which inhibits liver regeneration	Partial hepatectomy	(107)

*KCs, Kuppfer cells; MoMϕs, Monocytes-derived macrophages; LSECs, liver sinusoidal endothelial cells; HSCs, hepatic stellate cells; NKT cells, natural killer T cell; IFN-γ, Interferon-gamma; CCL2, chemokine (C-C motif) ligand 2; TNF-α, tumor necrosis factor alpha; TGF-β, transforming growth factor beta; IL-17, interleukin 17; IL-1β, interleukin 1 beta; IL-6, interleukin 6; IL-10, interleukin 10; ROS, reactive oxygen species; IDO, indoleamine 2,3-dioxygenase; PGE2/J2, prostaglandin E2/J2; MCP-1, monocyte chemoattractant protein-1; ICC, intrahepatic cholangiocarcinoma; LPS, lipopolysaccharides; HBV, hepatitis B virus; I/R, ischemia/reperfusion*.

### Crosstalk Between Hepatic Macrophages and Hepatocytes

The crosstalk between hepatic macrophages and hepatocytes has been indicated in a number of studies. In a mouse model of thioacetamide (TAA)–induced intrahepatic cholangiocarcinoma, KCs accumulate around the central vein area and express Notch ligand Jagged-1 rapidly after the initiation of the TAA treatment, coinciding with the activation of Notch signaling in pericentral hepatocytes. Depletion of KCs prevents the Notch-mediated hepatocytes transformation to cholangiocytes, suggesting that KCs contribute to the cell fate change in hepatocytes (109). Another study has also demonstrated that KCs are required for the proliferation of liver progenitor cells that can differentiate into hepatocytes in a model of choline-deficient, ethionine-supplemented diet–mediated liver injury and regeneration (110). In ALD, it is shown that KC-produced IL-6 triggers hepatocyte senescence, which becomes resistant to apoptosis (81). Interestingly, recent studies also support that hepatocytes communicate with macrophages through the release of extracellular vesicles (EVs) that contain proteins and microRNAs (82, 83). *In vitro* studies using HepG2 cells have demonstrated that alcohol treatment induces an elevated release of EVs, which activate THP-1 cells, a human leukemia monocytic cell line into a proinflammatory phenotype through CD40 ligand (83). Another study also showed that exosomes derived from alcohol-treated hepatocytes mediated the transfer of liver-specific miRNA-122 to monocytes and sensitized monocytes to LPS stimulation (82). These studies suggest that hepatocytes release EVs that contain altered proteins and miRNAs to regulate the activation of monocytes/macrophages.

### Interactions of Hepatic Macrophages With Cholangiocytes, HSCs, and LSECs

Macrophages secrete IL-6 during infection, and IL-6 can induce cholangiocyte proliferation leading to ductular reaction (84, 85). On the other hand, cholangiocytes are the major source of osteopontin and macrophage chemoattractant protein 1, which acts as chemotaxis to recruit MoMϕs during partial hepatectomy (102). Hepatic stellate cells and KCs are located in close proximity to each other (86, 103). In a mouse model of CCl_4_-induced liver fibrosis, it is shown that depletion of hypoxia-inducible factor 1α in HSCs inhibits KC activation and reduces the release of proinflammatory cytokines, suggesting a function of HSCs in regulating KCs during liver fibrosis (87). On the other hand, KCs have also been reported to modulate HSC functions. Chemokine (C-X-C motif) ligand 6 stimulates the phosphorylation of epidermal growth factor receptor and the expression of TGF-β in KCs, which further activates HSCs and results in liver fibrosis (103). It is reported that ROS and IL-6 activate KCs, which in turn modulate fibrogenic responses of HSCs (104). Activated KCs secrete interferon γ, which subsequently induces HSC apoptosis in a STAT1-dependent manner and reduces liver fibrosis (111). Liver sinusoidal endothelial cells are the major source of intercellular adhesion molecule 1 (ICAM-1). In partial hepatectomy, ICAM-1 expressed on KCs and LSECs recruits leukocytes, which leads to TNF-α and IL-6 production, thereby promoting hepatocyte proliferation (89). Moreover, MoMϕs also play an important role in activating LSECs and contributing to vascular growth and liver regeneration (88). Kupffer cell depletion inhibits hyaluronic acid uptake by LSECs and impairs sinusoidal integrity, suggesting there is a crosstalk between KCs and LSECs (37, 112).

### Interactions of Macrophages With Other Hepatic Immune Cells

Kupffer cell activation by pathogens results in CCL2 secretion, which promotes the recruitment of monocytes into the injured liver (105). It has been reported that alcohol treatment of THP-1 cells or human primary monocytes triggers the secretion of EVs, which induce the differentiation of naive monocytes into anti-inflammatory macrophages by delivering cargos, such as miR-27a (90).

Neutrophils are the most abundant white blood cells in the circulation, and they are recruited to the liver in various injury conditions (91). During cholestatic liver injury, neutrophils secrete IL-17, which promotes the production of TNF-α and TGF-β by KCs. On the other hand, KCs express adhesion molecules that induce neutrophil attachment and facilitate bacterial clearance in the liver (113). Another study reported that KCs coordinate with LSECs for neutrophil adhesion, recruitment, and activation through TLR4 during LPS-induced liver injury (78).

Natural killer T (NKT) cells are a group of “unconventional” T cells that express both natural killer (NK) cell receptors and T cell receptors. In the NASH liver, lipid accumulation causes an increase of the number of MoMϕs and activates the cells to a proinflammatory phenotype. Activated MoMϕs promote NKT cell activation and induce NKT cell deficiency, contributing to the pathogenesis of NAFLD (93). The importance of KC/NKT interaction in liver regeneration has also been described. After partial hepatectomy, MoMϕs produce IL-12 to activate hepatic NKT cells, which prohibit liver regeneration (114). In ALD, NLRP3 inflammasome activation results in IL-1β release by KCs. Released IL-1β recruits and activates hepatic invariant NKT cells, which promote liver inflammation, neutrophil infiltration, and liver injury (106). Furthermore, ample evidence supports the importance of macrophages in interacting with conventional T cells. It has been shown that KCs trigger the recruitment of CD4^+^ T cells by ROS, IL-6, and TNF-α (107). Kupffer cells express high levels of MHC class II and act as antigen-presenting cells. In fact, a number of studies have shown that KCs play an important role in inducing and maintaining T cell tolerance, through producing tolerogenic mediators such as IL-10, TGF-β, ROS, indoleamine 2,3-dioxygenase, prostaglandin E_2_/J_2_ (94, 97). Moreover, it is reported that during liver transplantation KCs induce T cell apoptosis and thus promote immune tolerance (98). After HBV infection, KCs produce IL-10 and support liver tolerance by inducing anti-HBV CD8^+^ T cell exhaustion (99). A recent study demonstrated that the induction of endoplasmic reticulum stress in HCC cell lines induces the secretion of EVs. The HCC-derived EVs induce elevated expression of programmed death ligand 1 on macrophages *in vivo* and *in vitro*, which causes T cell dysfunction and impaired proliferation (100). Recent studies showed that priming CD8^+^ T cells by KCs leads to the differentiation of CD8^+^ T cells into effector cells that have powerful killing activities against hepatitis viruses, such as HBV (101).

Moreover, interesting interactions between macrophages and platelets have been unveiled (115). Under basal conditions, platelets form transient “touch-and-go” interactions with von Willebrand factor constitutively expressed on KCs (115). During bacterial infection, platelets switch from “tough-and-go” to sustained GPIIb-mediated adhesion on the KCs to encase the bacteria and facilitate anti-bacterial responses (115).

In summary, hepatic macrophages interact with almost all cell types of the liver. In naive state, KCs, through these interactions, play a critical role in maintaining tissue homeostasis. Under injury or disease conditions, the crosstalk of macrophages with hepatocytes, cholangiocytes, LSECs, HSCs, and other immune cells contributes to exacerbation of tissue damage or promotion of liver repair and regeneration, depending on the cell type and signaling pathways involved.

## Author Contributions

All authors listed have made a substantial, direct and intellectual contribution to the work, and approved it for publication. ZS wrote the draft. CJ revised the manuscript.

## Conflict of Interest

The authors declare that the research was conducted in the absence of any commercial or financial relationships that could be construed as a potential conflict of interest.
